# Functional Restriction for the Fear of Falling In Family Caregivers

**DOI:** 10.1097/MD.0000000000001090

**Published:** 2015-07-13

**Authors:** Jing Shen, Fangke Hu, Fucun Liu, Peijian Tong

**Affiliations:** Orthopedic Department, The First Affiliated Hospital of Zhejiang Chinese Medical University, Zhejiang Provincial Hospital of TCM, Hangzhou (JS, FL, PT); Orthopedic Department, Tianjin Hospital, Tianjin, PR China (FH).

## Abstract

Hip fractures often result from falls, and most family caregivers fear another fall. This study aimed to assess this fear in family caregivers and analyze its influence on functional recovery.

This study was retrospectively performed by interview at the clinic or through telephone contact. The Falls Efficacy Scale International (FES-I) was used to assess fall-related feelings of patients and their family caregivers.

Of the 539 patients studied, hip fracture was caused by a fall in 467 (86.6%). The mean FES-I value of the family caregivers was significantly lower than that of the patients (85.39 versus 99.02, *P* < 0.001). The mean patient functional recovery score (FRS) was 68.41. A fracture caused by a fall and recurrent fall-related fractures both reduced caregiver FES-I scores. The difference between patient and caregiver FES-I scores showed a significant positive correlation with the FRS (*P* < 0.001).

Family caregivers were more concerned about falls than were patients. Furthermore, a greater difference in the fall-related reaction between caregivers and patients was associated with greater adverse effects on rehabilitation.

## INTRODUCTION

The incidence of hip fracture increases with age.^1,2^ Hip fracture is a major cause of morbidity and mortality in the elderly, and most hip fractures result from a fall.^3,4,5,6,7^ Two-third of elderly people who survive a hip fracture require walking aid for at least 1 month, and more than half continue to experience restricted activities of daily living after 1 year.^8^ Many factors influence functional recovery after hip fracture repair.^9^ Fear of falling (FOF) is one of the most significant factors affecting functional recovery after hip fracture in elderly people.^10^ Elderly individuals with a FOF walk less because of hip weakness and have a slower walking speed.^11^ The decreased physical activity further weakens muscles and has negative effects on strength, physical function, and socialization. This, in turn, increases FOF, inciting a vicious circle of greater FOF leading to more falls.^12^ Activity restrictions because of FOF may negate any benefit of rehabilitation, which could limit the long-term success of rehabilitation programs after hip fracture.^13^

During the long recovery period after hip fracture surgery, 89% of these patients’ caregivers are immediate family members, offspring, and spouses.^8^ Evidence shows that family caregivers have a constant fear of their loved one falling and becoming more dependent on them after a fall.^14^

We inadvertently found that some family members were more concerned about the recovery than the patients, which had a seemingly harmful effect on the patients’ recovery. Few studies have investigated the influence of family caregivers’ FOF on recovery; as such, this study aimed to reveal FOF severity in family caregivers.

## PATIENTS AND METHODS

**FIGURE 1:**
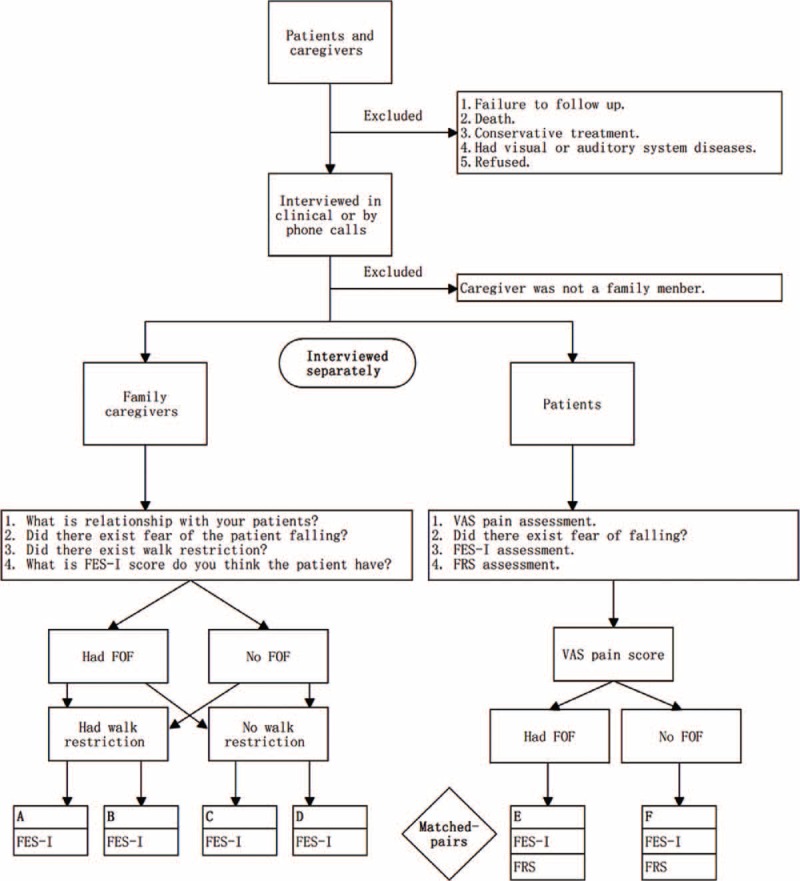
The follow-up flowchart. A, B, C and D were groups of family caregivers, E and F were groups of patients. Group A: family caregivers had FOF and walk restriction. Group B: family caregivers had no FOF but had walk restriction. Group C: family caregivers had FOF without walk restriction. Group D: family caregivers had no FOF and restriction. Group E: patients had FOF. Group F: patients had no FOF. FES-I = The Falls Efficacy Scale International; FOF = fear of falling; FRS = functional recovery score; VAS = visual analog scale for pain.

The medical records of hip fracture patients >60 years of age treated in our institute between January 2007 and December 2009 were reviewed. The exclusion criteria were as follows: failure to complete follow-up, death, nonoperative treatment, visual or auditory system disease, and refusal. If caregivers were not family members, they were excluded from the follow-up process. The follow-up flowchart is shown in Figure 1.

Patient data obtained from medical records included age, sex, cause of fracture, secondary surgery for the fracture, medical history (diabetes, hypertension, coronary heart disease, dementia, tumor, and chronic obstructive pulmonary disease), ambulation status prefacture (independent or dependent), postoperative complications (cardiac events, pulmonary infection, venous thromboembolism, cerebral infarction, and gastrointestinal bleeding), length of hospital stay, and admission to a rehabilitation facility postdischarge. The Falls Efficacy Scale International (FES-I), which has demonstrated reliability and validity^15^ was chosen to assess FOF in family caregivers and patients. Hip function recovery was assessed by the functional recovery score (FRS).^16^ The visual analog scale (VAS) was used to evaluate hip pain. Data on the FES-I score, FRS, VAS score, rehabilitation location, secondary fall, and secondary fall fracture after discharge were obtained via a clinical or telephone interview. The family caregivers and patients were interviewed separately (Figure 1). Postdischarge falls and secondary fall fracture were also assessed in the interview. Then family caregivers and patients were matched for the further statistical analysis.

Paired *t-*tests or related Wilcoxon rank sum tests were used to compare numerical variables between matched-pair groups. Pearson correlation analysis or curve estimation was used to estimate correlations between 2 variables. A 1-way analysis of variance or Kruskal-Wallis test was used to compare multiple groups. Multiple linear regression analyses were also used to evaluate the relationship between risk factors and dependent variables (dependent variable would be transformed by a rank case calculation [Blom's formula] if not normally distributed). Residual analyses were performed to check regression model assumptions. *P* values < 0.05 were considered statistically significant. The statistical analyses were performed using SPSS version 13.0.

The ethics committee approved the study and each participant provided verbal consent.

## RESULTS

A total of 655 elderly patients with hip fracture were reviewed. Of them, 116 were excluded for failure to complete follow-up (N = 10), death (N = 75), nonoperative treatment (N = 6), visual or auditory system disease (N = 8), refusal (N = 5), and absence of family caregivers (N = 12); therefore, 539 patients (172 men [31.9%], 367 women [68.1%]; mean age, 76.98 years) were ultimately interviewed in this study. The median follow-up period was 24 months (range, 12–35 months). The patients’ basic characteristics are shown in Table 1.

**Table 1:**
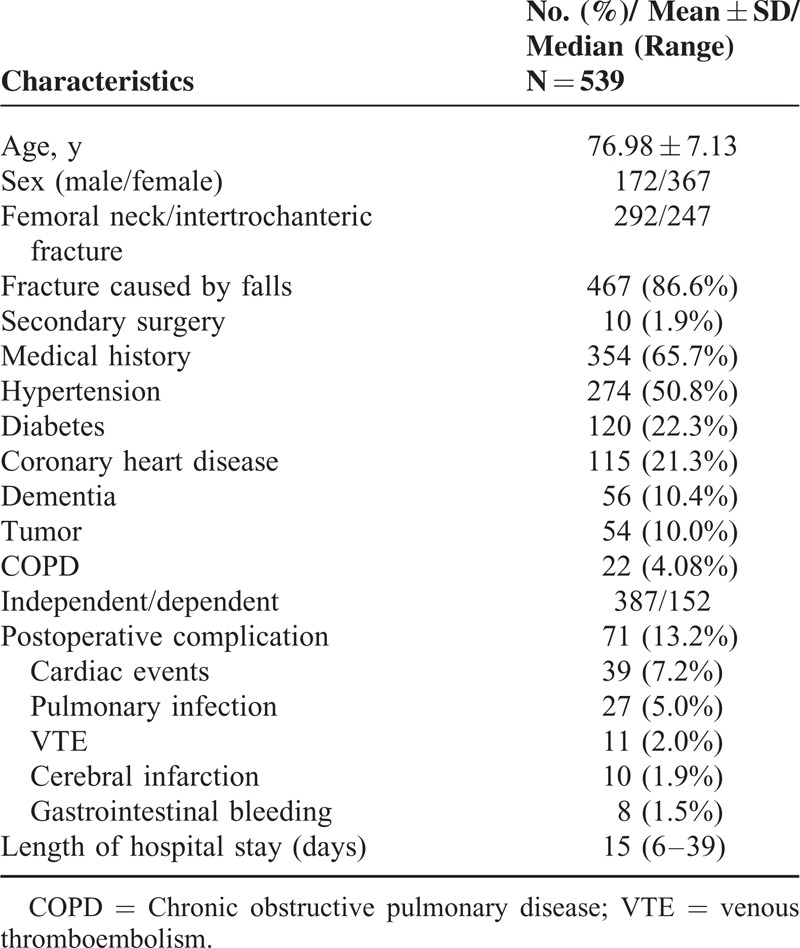
Characteristics of Hip Fracture Patients

**FIGURE 2:**
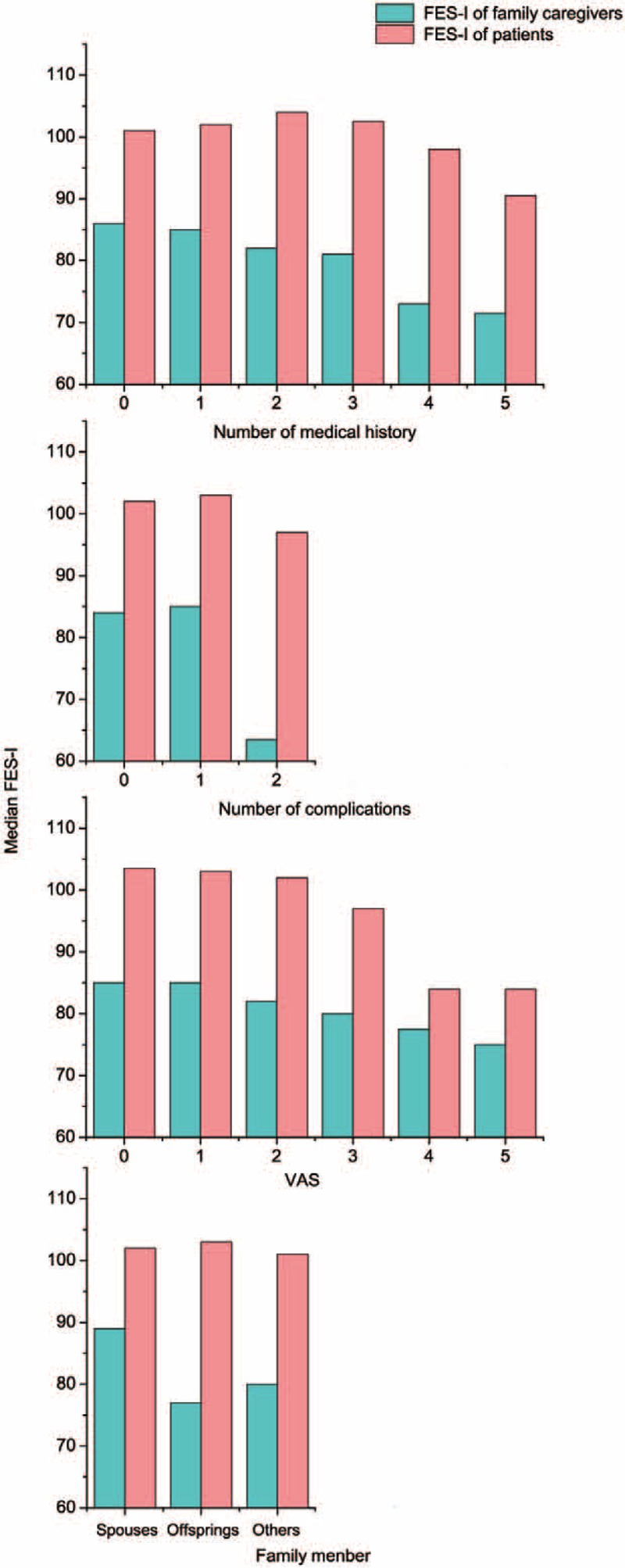
Median FES-I of multiple categorical variables. FES-I = The Falls Efficacy Scale International.

The 492 (91.3%) immediate family caregivers included 321 spouses and 171 offsprings. Of the study population, 396 (75.4%) family caregivers and 381 (70.7%) patients showed FOF. A total of 72 (13.4%) patients had falls postdischarge, including 39 (7.2%) with a fracture and 32 (5.9%) with a secondary fall fracture. Forty-one (7.6%) patients were admitted to a rehabilitation facility postdischarge, whereas 316 (58.6%) patients reported pain in the injured leg. The median FES-I values of the family caregivers and patients were 84 (range, 30–137) and 102 (range, 26–129), respectively, demonstrating a significant difference (z = − 12.00, *P* < 0.001, two-tailed). The median FES-I values of the family caregivers’ and patients’ characteristics by binary classification are shown in Table 2, whereas the median FES-I values of multiple categorical variables are shown in Figure 2. The median patient VAS score and FRS were 1 (range, 0–5) and 76 (range, 5–100).

**Table 2:**
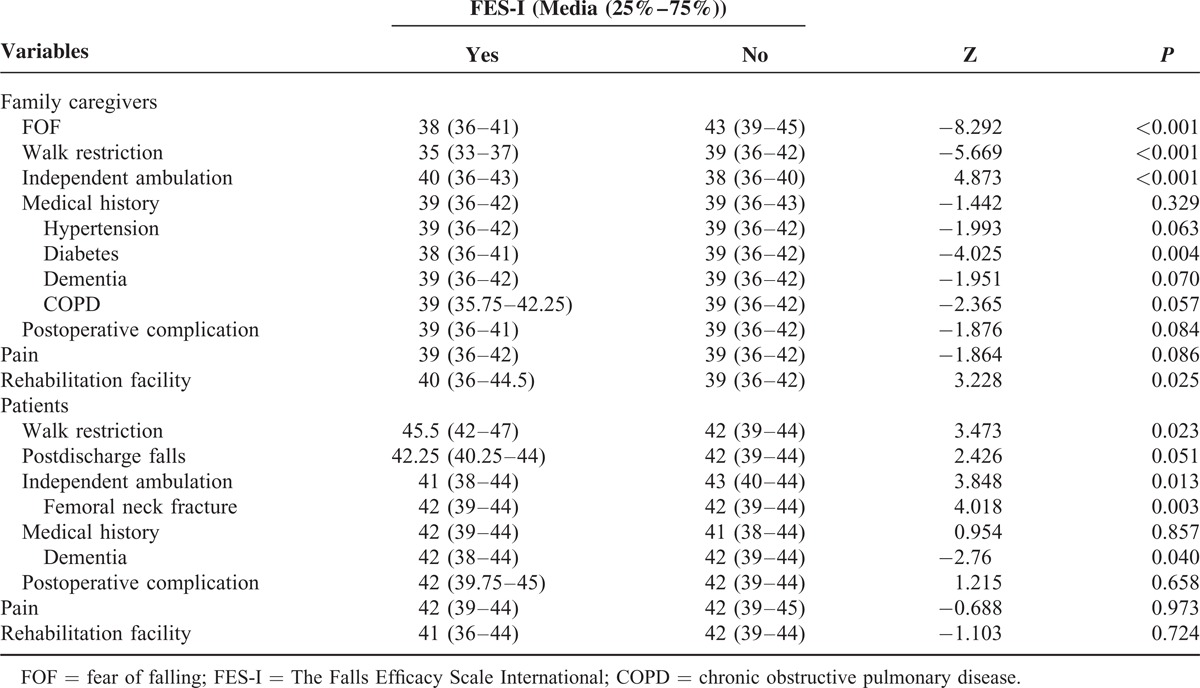
FES-I Score of Multiple Categorical Variables

After the family caregivers were matched with the patients, the numbers were distributed from the A to F groups, and the FES-I score, VAS score, and FRS are shown in Table 3. Thirty-six (6.7%) family caregivers reported walking restrictions, of which 6 denied having FOF.

**Table 3:**
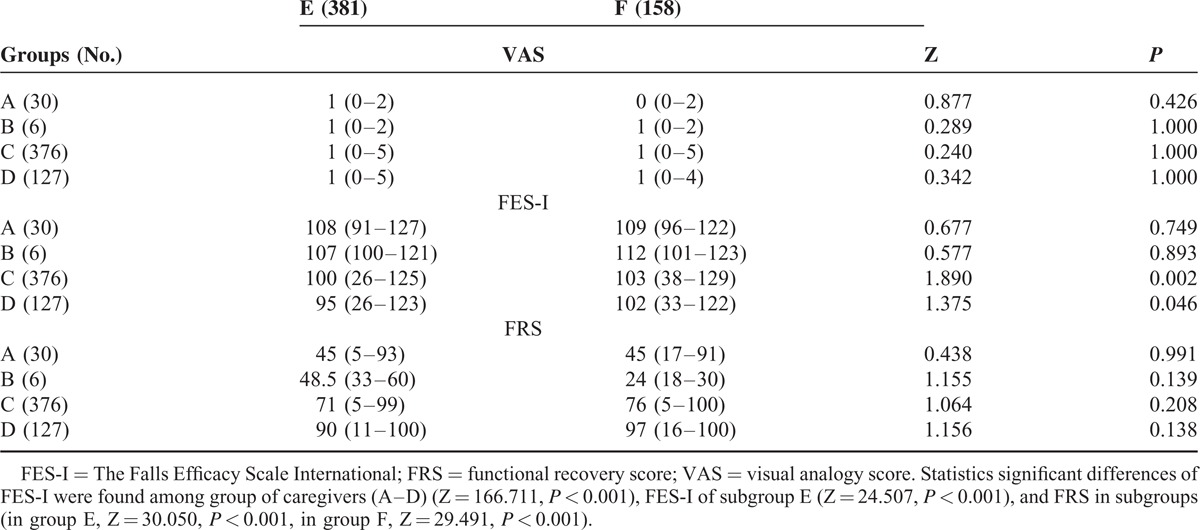
Difference of VAS, FES-I, FRS Among Groups

**FIGURE 3:**
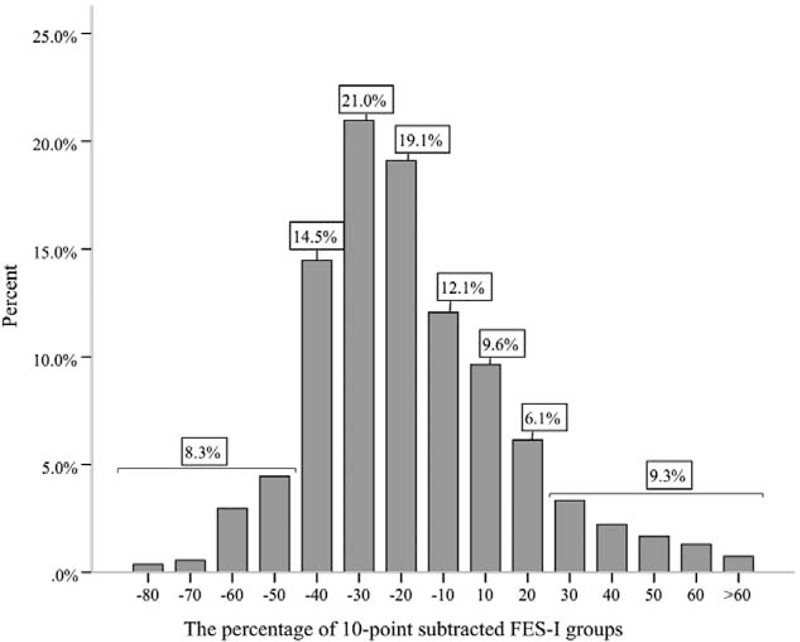
Percentage of the total sample in each 10-point subtracted FES-I groups. FES-I = The Falls Efficacy Scale International.

The difference between the patients’ and family caregivers’ FES-I values (hereafter, subtracted FES-I score) was −17 (range, −79 to 74) points. A total of 116 (21.5%) family caregivers had a higher FES-I score than their matched patients, 9 (1.7%) were on the same level, and 414 (76.8%) were lower. The total samples were divided into groups based on 10-point increments of the subtracted FES-I. The percentages of the total samples in each 10-point subtracted FES-I group are shown in Figure 3.

**FIGURE 4:**
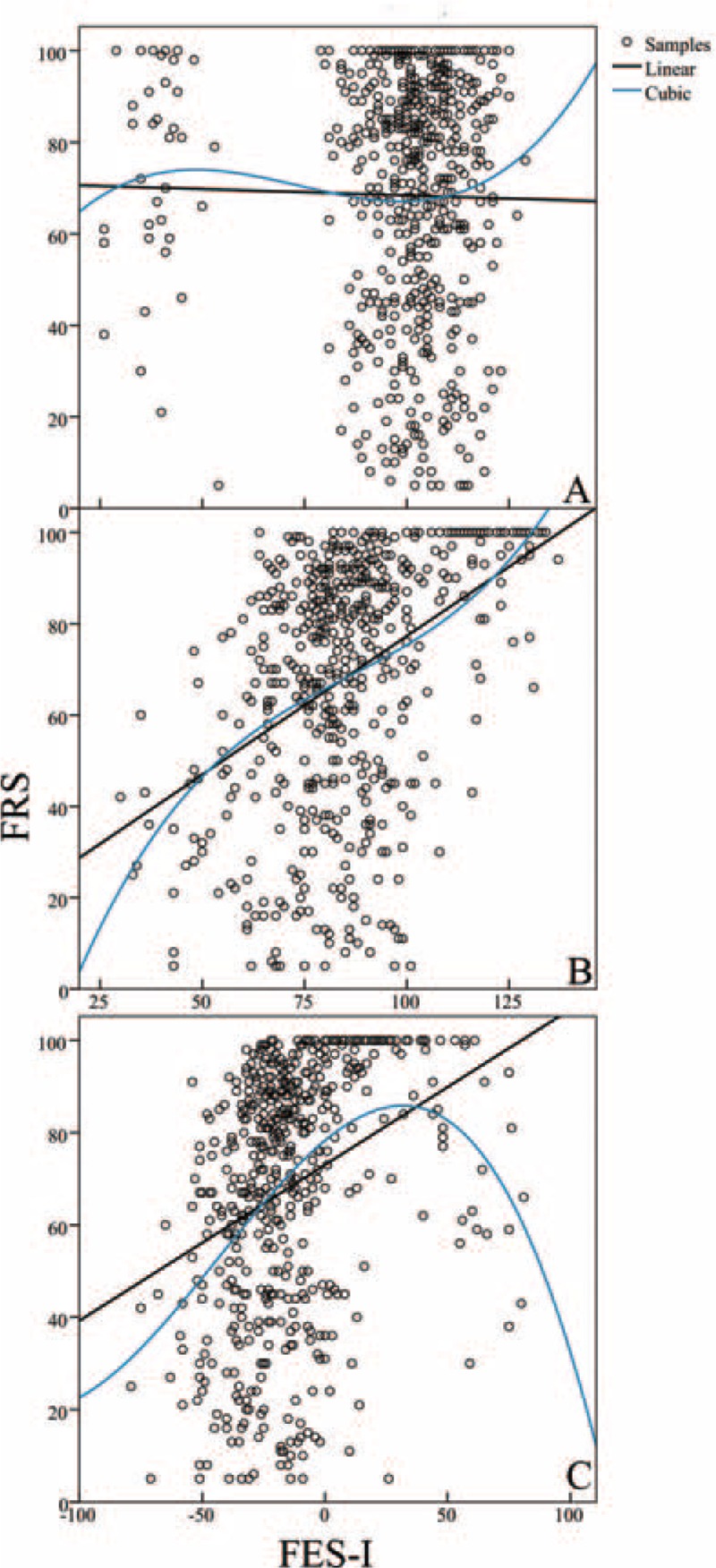
Curve fitting between FES-I and FRS. Linear (black line) and cubic (blue line). Curve estimation models were shown as follows: A, Between FES-I of patients and FRS. *R*^2^ in each model was 0.000 and 0.005 (*P* > 0.05). B, Between FES-I of family caregivers and FRS. *R*^2^ in each model was 0.180 and 0.184 (*P* < 0.001). C, Between subtracted FES-I and FRS. *R*^2^ in each model was 0.105 and 0.154 (*P* < 0.001). FES-I = The Falls Efficacy Scale International; FRS = functional recovery.

Curve estimation was performed to estimate the correlation between FES-I score and FRS (Figure 4), and the fitting line was estimated by multiple models. The optimum fitting line between the FES-I score of the family caregivers and the FRS showed a cubic model (Figure 4B, blue line, *R*^*2*^ = 0.184, *P* < 0.001), whereas the subtracted FES-I score and FRS showed a cubic model (Figure 4C, blue line, *R*^*2*^ = 0.154, *P* < 0.001). There, however, was no optimum fitting line between the patients’ FES-I score and their FRS.

Multiple linear stepwise regression analyses (case labeled by patient identification) were used after FRS was transformed. Five variables were ultimately entered into a regression model (Table 4).

**Table 4:**
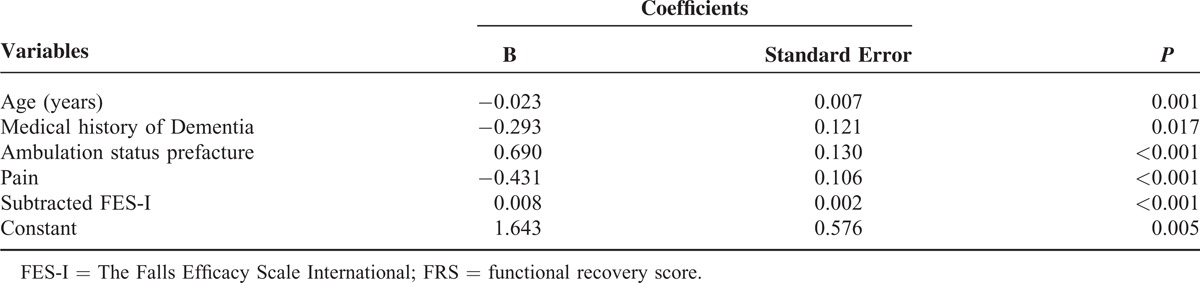
Results of Multivariate Regression Analysis Between Risk Factors and FRS

## DISCUSSION

We attempted to assess the FOF in family caregivers and used the FES-I in a novel way. One study investigated the discordance in FOF between family caregivers and elderly hip fracture patients. The FES-I score was used to assess the FOF in caregivers and patients with total FES-I scores of 99.02 ± 18.47 and 85.39 ± 18.91, respectively. The caregiver FES-I score was significantly lower (*P* < 0.001, Figure 1) than the patient FES-I score, and the subtracted FES-I score was −30 to −12 points (Figure 2). Although references to FOF by family caregivers have been made in the literature,^14^ to the best of our knowledge, this is the first assessment of FOF among family members who are caring for an elderly patient with a hip fracture.

Patient FOF has a negative impact on postoperative functional exercise.^17^ Oude^10^ suggested that FOF has a larger impact than many other harmful factors.^18^ Elderly hip fracture patients nearly always experience FOF, which reduces their physical activity.^10^ Here we found a mean FES of 68.41 ± 27.05 points. The results also showed a positive correlation between the subtracted FES-I score and FRS (Figure 3), indicating that a greater discrepancy between caregivers’ and patients’ perceptions negatively impacts functional recovery. Our results further suggest that caregiver FOF has a greater influence on functional recovery. For this reason, reducing the patient FOF^10^ cannot sufficiently improve functional recovery; caregiver FOF must also be reduced.

In this study, 86.6% of hip fractures were caused by a fall. Falls have been shown to cause nearly 90% of hip fractures in patients aged >65 years.^7^ A simple fall is more likely to result in a hip fracture in elderly individuals because of the impaired protective response, muscle weakness, and, more importantly, the presence of osteoporosis.^6^ Among hip fracture patients, 21% to 85% experienced FOF,^19^ and that number increases significantly in elderly patients after a fall.^20^

A previous study found that a prior fall was one of the main factors leading to FOF.^19^ Our results showed that a fracture resulting from a fall was likely to increase FOF in both caregivers and patients. The mean FES-I score after a fall fracture in caregivers and patients was 84.36 ± 18.07 and 98.43 ± 19.45 points, respectively, which was significantly lower than that in patients who had not experienced a fall fracture. This difference was associated with a lower FRS. Thus, a fall fracture postdischarge had significant negative effects on FOF in caregivers and on the FRS.

Some methods effectively decrease FOF and lessen the risk of fall after hip surgery, including the use of a hip protector,^21^ early discharge and home-based rehabilitation,^22^ and intensive physical therapy,^23^ although these methods may not show quick results.^24^ The effectiveness of these interventions in terms of their impact on family caregivers, however, has not been studied. The study showed that scheduled reexaminations significantly improved both the caregiver FES-I score and the FRS (Figure 4). At each follow-up examination, a surgeon checked bone healing radiographically and made appropriate recommendations for rehabilitation. After the third follow-up visit, the mean difference in FES-I score between caregivers and patients decreased from 38.11 to 10.71 points, whereas the FRS increased from 35.22 to 75 points.

Some limitations of this study should be mentioned. First, the retrospective design and use of telephonic interviews introduce the possibility of recall bias. Second, prefracture FOF could not be assessed. Finally, factors influencing caregiver FOF were not fully explored and need further study.

## CONCLUSIONS

If the patient's formal rehabilitation program (in a facility) is insufficient, then the availability of a family caregiver is critical. Patients’ daily activities primarily depend on their caregivers’ decisions. Family caregivers were more concerned about a fall than the patients. Furthermore, the greater the difference between patient and caregiver FOF, the greater was its negative effect on rehabilitation. Regular follow-up examinations showed a lower family caregiver FES-I score and a higher patient FRS. This study's findings indicate that family caregivers’ concerns must be considered in the planning of a rehabilitation program.
